# A Didactic Model of Macromolecular Crowding Effects on Protein Folding

**DOI:** 10.1371/journal.pone.0011936

**Published:** 2010-08-03

**Authors:** Douglas Tsao, Allen P. Minton, Nikolay V. Dokholyan

**Affiliations:** 1 Department of Chemistry, University of North Carolina, Chapel Hill, North Carolina, United States of America; 2 Section on Physical Biochemistry, Laboratory of Biochemistry and Genetics, National Institute of Diabetes and Digestive and Kidney Diseases, National Institutes of Health, United States Department of Health and Human Services, Bethesda, Maryland, United States of America; 3 Department of Biochemistry and Biophysics, University of North Carolina School of Medicine, Chapel Hill, North Carolina, United States of America; Griffith University, Australia

## Abstract

A didactic model is presented to illustrate how the effect of macromolecular crowding on protein folding and association is modeled using current analytical theory and discrete molecular dynamics. While analytical treatments of crowding may consider the effect as a potential of average force acting to compress a polypeptide chain into a compact state, the use of simulations enables the presence of crowding reagents to be treated explicitly. Using an analytically solvable toy model for protein folding, an approximate statistical thermodynamic method is directly compared to simulation in order to gauge the effectiveness of current analytical crowding descriptions. Both methodologies are in quantitative agreement under most conditions, indication that both current theory and simulation methods are capable of recapitulating aspects of protein folding even by utilizing a simplistic protein model.

## Introduction

Current information about the relative stability of native and non-native conformations of proteins is derived largely from experiments carried out on dilute solutions of protein subjected to variations in temperature and pH, or addition of chaotropic agents or osmolytes [1], [2], [3]. Yet most proteins exist *in vivo* within local environments containing a high total weight to volume concentration of proteins and other macromolecules [4], [5]. Such excluded volume environments can directly affect and alter a protein's function by inducing conformational changes [6], [7], [8], or potentially enhance the formation of aggregate species [9].

Thermodynamic considerations dictate that the stability of a particular protein in dilute solution is directly linked to the difference between the respective free energies of nonspecific interaction of the native and non-native conformations of the selected protein and the macromolecular constituents of the surrounding medium [10], [11]. Although such interactions may be of any kind, in the present work we focus upon intermolecular excluded volume interactions, or steric repulsions. Such interactions are ubiquitous in highly volume-occupied physiological fluid media, and result in significant size- and shape-dependent repulsive contributions to the chemical potential of each macromolecular species that tend to stabilize more compact conformations relative to less-compact conformations [12], [13]. In this context we shall refer to macromolecules in the environment interacting with target protein via steric exclusion as “crowders”.

Previous studies of the effect of volume exclusion upon protein stability have fallen into one of two categories: (1) In statistical thermodynamic models [14], [15], [16], [17] the effect of volume exclusion is treated as a conformation-dependent potential of mean force acting between crowding molecules (or equivalent hard particles) and the tracer molecule in either native or non-native conformations; (2) In atomic or coarse-grained simulations [18], [19], [20], volume exclusion is incorporated explicitly into the simulated system, which consists of a single tracer molecule capable of undergoing conformational transitions and a substantial number of rigid crowder molecules (or hard particles) occupying a specified fraction of total volume. Monte-Carlo or Brownian Dynamics simulations are then performed to elucidate the equilibrium and time-dependent behavior of the system.

Although statistical-thermodynamic models offer quantitative estimation of the effect of crowding on protein stability, descriptions of the potential of mean force acting between a rigid crowding particle and a flexible non-native protein conformation remain highly approximate, and the estimates of the magnitude of crowding effects on conformational equilibria vary widely [15], [21]. Molecular dynamics simulations permit studies of currently theoretically-untreatable systems, such as a solution containing multiple species of crowding molecules that interact with each other via non-additive potentials of mean force, and testing of theoretical approximations [22]. Recent efforts to expedite calculation times have utilized trajectories derived from proteins simulated with molecular dynamics to calculate its chemical potential from an analytically derived distribution of hard spheres [23], [24]. However, conventional molecular or atomic-level Brownian dynamics calculations are at present too computationally-intensive to permit thorough exploration of the effects of numerous variables. The use of coarse-grained models have served as a useful alternative in simplifying computation times while yielding new insights into crowding effects at the residue-level [25], [26], [27]. Coarse-grained models have become even more sophisticated with the emergence of virtual cytoplasms simulating the presence of many different protein species [28], [29].

Discrete molecular dynamics (DMD) has recently been used as another alternative for studying the effect of macromolecular crowding on conformational equilibria [30], [31]. DMD is a rapid method of simulation in which interactions between particles are described by step-wise or “histogram” potentials. In DMD, simulations proceed according to ballistic equations of motion and velocity-modifying events are sorted through use of a search algorithm. The combined usage of simplified models and the intrinsic DMD algorithm has led to simulations at biologically-relevant timescales with reduced simulation times [32]. Other uses of DMD encompass studies in protein folding [33] and aggregation [34], as well as RNA [35], protein-DNA complexes [36], and lipids [37].

At its most fundamental level, protein folding is a process whereby different parts of a heteropolymeric chain that are initially separated in space come together to form a highly compact specific structure that could be described as a condensed microphase. The interaction between two chain elements (amino acids) consists of two parts: a short-ranged direct interaction, such as an electrostatic, hydrophobic or hydrogen bonding interaction between two side chains, and a longer range indirect interaction imposed by the covalent linkage between the two amino acid residues. We propose as a didactic aid to understanding the interplay between these contributions the simplest possible model that contains both types of interactions, and investigate the effects of its folding equilibria upon the addition of crowders.

In this model, a “protein” consists of two rigid spherical subunits interacting by a one-dimensional potential that is the sum of direct and indirect contributions. The direct interaction is represented by a very short-ranged square well potential, the depth of which is parameterized to vary with the concentration of a chaotropic agent, urea, so as to mimic the experimentally measured dependence of the two state unfolding of a simple protein upon urea concentration. The indirect interaction is represented by a longer ranged empirical function of inter-subunit distance, parameterized to mimic the distribution of radii of gyration of an unfolded protein calculated from a detailed atomic-level simulation. Thus this primitive model exhibits certain features of the behavior of actual proteins while retaining simplicity of calculation that allows its properties to be explored in depth, both analytically and with the aid of dynamic simulation. In the following section we describe the model. Next, we present the statistical-thermodynamic description of the model, followed by details of the DMD simulations. Then results of each method of calculation are presented and compared. We find that the two approaches are in semi-quantitative agreement under most conditions.

## Methods

### One-dimensional model for two-state protein folding at constant temperature in the presence of varying concentrations of urea

**Figure 1:**
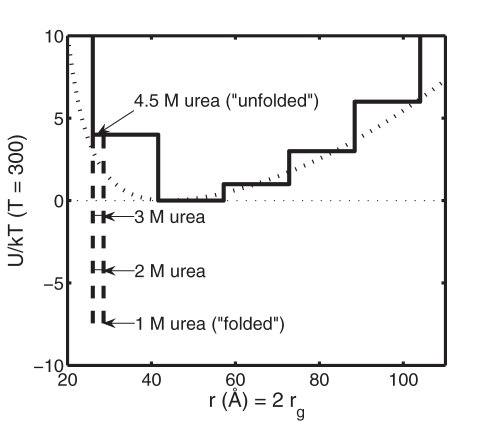
Histogram potential for one-dimensional toy model. Solid curves: discretized potential acting between two isolated spherical protein “subunits” as a function of the distance between sphere centers (*r = r_g_*/2). Solid line: potential in the presence of 4 M urea. Dashed lines: attractive potential in bin 1 (see Table 1) calculated for *c_urea_* = 1, 2 and 3 M. Dotted curve: continuous potential of mean force as a function of *R_g_*, derived from the distribution of *R_g_* of unfolded states of ribonuclease A calculated by Goldenberg [38] as described in Minton [15].

A “protein” consists of two hard spherical “subunits” of radius 13 Å (subsequently referred to as subunit spheres) interacting via a potential, specified in Table 1 and plotted in Fig. 1, that depends only upon the distance between the centers of the two subunit spheres, denoted by *r*. In this model, the radius of gyration of the protein, denoted by *r_g_*, is simply *r*/2. The potential consists of three parts: a hard repulsive core defining the distance of close contact between the two subunit spheres, a urea-dependent short-ranged potential (bin 1) representing the compact native conformation of the protein, and a longer-ranged urea-independent potential (bins 2–6) representing the manifold of non-native conformations.

**Table 1:** Specification of potential of average force *U_0_* acting between spherical protein “subunits” in the absence of crowding particles.

Bin	*r*	*U_0_/kT*	Conformation
	*<r_0_*	∞	inaccessible
1	*r_0_–r_1_*	−10.8+3.3 c_urea_	“Native”
2	*r_1_–r_2_*	4	“Non-native”
3	*r_2_–r_3_*	0	
4	*r_3_–r_4_*	1	
5	*r_4_–r_5_*	3	
6	*r_5_–r_6_*	6	
	>*r_6_*	∞	inaccessible

The boundaries between histogram bins are situated at the following values of *r* (Å): *r_0_* = 26, *r_1_* = 28.6, *r_2_* = 41.6, *r_3_* = 57.2, *r_4_* = 72.8, *r_5_* = 88.4, *r_6_* = 104.

This potential was designed to emulate certain properties of ribonuclease A, namely: (a) The size of the two protein subunit spheres was chosen so that the radius of gyration of two tangent subunit spheres (the “native” state) matches that of native ribonuclease A [38]. (b) The non-native potentials were chosen to provide an equilibrium distribution of *r_g_* (calculated as described below) qualitatively resembling that calculated for unfolded ribonuclease A by Goldenberg [38]. (c) The dependence of the contact potential upon urea concentration was chosen so that the resulting dependence of the equilibrium fraction of natively folded protein upon urea concentration, as calculated in the model in the absence of crowder, would resemble that observed experimentally for ribonuclease A [39].

### Statistical thermodynamic calculation of the equilibrium properties of the one-dimensional protein model

The equilibrium probability of occurrence of the state with r = r* at temperature *T* is given by
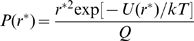
(1)where
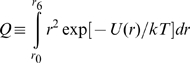
(2)and the equilibrium fraction of protein residing in the *i*th bin (as defined in Table 1) is given by
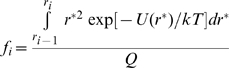
(3)The fraction of model protein in the “native” state (i.e., bin 1) then becomes
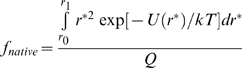
(4)


### Statistical-thermodynamic model for crowding by hard spheres

The statistical-thermodynamic model for protein stability described above is generalized to include the potential of average force acting upon the two subunit spheres in a fluid of hard spherical particles:
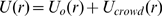
(5)where
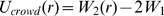
(6)Here *W_1_* is the work (in units of *kT*) associated with the insertion of a single hard sphere of radius *r_1_* into a hard sphere fluid containing a volume fraction *φ* of hard spheres of radius *r_c_*, and *W_2_(r)* is the work of insertion of a doublet of hard spheres of radius *r_1_* separated by distance *r* into the same fluid.

The scaled particle theory (SPT) initially developed by Reiss and coworkers [40] provides an approximate yet realistic means for calculating the free energy of creating a convex cavity with the dimensions of the particle to be inserted that contains no part of any other particle in the fluid. Thus SPT provides a direct means for evaluation of *W_1_* [41]. When the distance between the two subunit spheres exceeds a characteristic isolation distance *r_i_* = 2*r*_1_+2*r_c_*, the excess work of inserting two spheres is assumed to be twice the work required to insert a single sphere, because the cavities in the fluid required for insertion of both spheres do not overlap. However, when *r<r_i_*, SPT can no longer be used, since the cavities fuse and the joint cavity cannot be treated as a convex body [42]. We can, however, calculate the leading term in the expansion
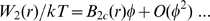
(7)from the statistical-thermodynamic relation
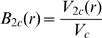
(8)where *V_2c_(r)* denotes the volume excluded by the two “protein” spheres with centers separated by distance *r* to the center of mass of the crowding sphere, and *V_c_* denotes the volume of the crowding sphere [43]. For *r* 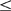 *r_i_*,
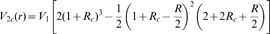
(9)where *V_1_* denotes the volume of a single “protein” sphere, and *R_c_* denotes the ratio 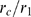, and *R* denotes the ratio *r/r_1_*.

We assume that for the purpose of calculating the work of cavity formation in a fluid of spherical crowders via SPT, the doublet of subunit spheres with *r* 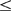 *r_i_* may be approximated by a single equivalent spherocylinder with diameter 2*r_1_* and a cylindrical length/diameter ratio *L_equiv_(r)* such that the co-volume of the equivalent spherocylinder with spherical crowder, denoted by *V_sc_*, is identical to that of the doublet of subunit spheres with separation *r*. Since
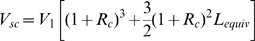
(10)the approximation of equal co-volumes leads to the relationship
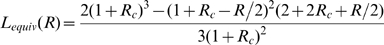
(11)where R denotes the ratio *r/r_1_*, over the range 0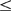 *R* 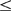 *2+R_c_*. Given the dimensions of the equivalent spherocylinder, the value of *W_2_(R)* may be estimated via the SPT expression for the work of insertion of a single spherocylinder into a fluid of hard spheres [44], [45]:
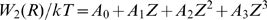
(12a)where
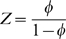
(12b)

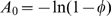
(12c)

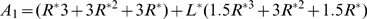
(12d)

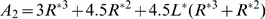
(12e)

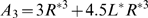
(12f)

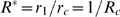
(12g)

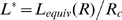
(12h)Note that *W_1_* = *W_2_*(*R* = 0), and in that limit, Eq. 12 is exact for all 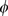. Moreover, due to the assumption embodied in Eq. 11, Eq. 12 is exact for all *R* 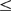
2+2*R_c_* in the limit 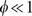.

### Discrete Molecular Dynamics

Discrete molecular dynamics differs from traditional simulation methods in that calculation of forces is discretized into intervals as opposed to the continuous calculation of forces [46], [47]. To accommodate the discontinuous nature of DMD simulations, step-wise potentials are used so that forces remain constant until two particles encounter a step in the potential [31], [32], [48]. Here we use the term event to denote an instance where two particles are within a defined interaction range. Simulations in DMD proceed as a series of two-body interactions, where the velocities of all particles in the system are evaluated within time intervals.

Let us consider a system of particles, including two particles *i* and *j* both of mass *m* that occupy initial positions of *r_i_*_0_ and *r_j_*_0_ with initial velocities of *v_i_* and *v_j_*. For simplicity we discuss a scenario where the interaction potential consists of one step, otherwise known as a square well potential, defined as
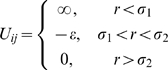
(13)The variable *σ_α_* defines an interaction distance, where *α* = 1 refers to the hard sphere repulsion and *α* = 2 is the attractive interaction. During a simulation, a table is generated by calculating event times for each pair of particles. The time interval in which an event may occur between two particles is
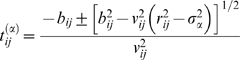
(14a)where
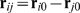
(14b)

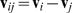
(14c)

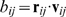
(14d)and the plus-minus sign refers to two particles either approaching or receding from each other, respectively. The trajectory of particle *i* is evaluated with respect to time as
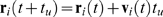
(15)where the time unit *t*_u_ is determined by comparing the shortest event time to the maximum allowed time interval *t_m_*. If 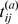 is greater than *t_m_*, then the particles are only permitted to move for *t_m_* and a new table is subsequently generated. Otherwise the shortest event time is the first considered, and the velocity changes according to the conservation laws of energy and momentum.

When particles *i* and *j* are determined to interact at 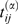, the squared difference in the particles' positions is compared to the squared interaction distance prior to a change in the potential (i.e., before 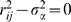). There are three possible scenarios for our one-step potential as the two particles approach one another. During an attractive encounter, if 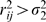 then the magnitude of separation between the particles decreases and the change in velocities will be
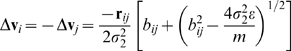
(16)However if 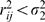, then the two particles increase their magnitude of separation and the change in velocities will be
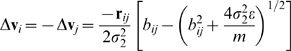
(17)In the case of hard sphere repulsions, the change in velocities is simply
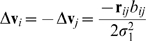
(18)


After each event, we remove from the table events that were calculated with the previous two particles since their velocities and positions have changed. The number of possible events with these two particles are then recalculated and sorted within the table. Simulations then proceed through the table, where particles are allowed to move between time intervals, until either 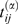
<*t_m_* or the table of events is depleted.

We incorporate the Andersen thermostat to simulate under canonical (constant *N*, *V*, *T*) conditions [49]. Temperature is maintained constant by surrounding the system of particles with a heat bath. The heat bath itself is comprised of imaginary ghost particles [31], [50] with number density *ρ_g_* that undergo stochastic collisions with the system via a Poisson process
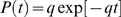
(19)Here *P*(*t*) represents the probability that a randomly chosen particle within the system undergoes a collision with a ghost particle at time *t*. The constant *q* represents the rate at which system particles undergo collision with the ghost particles. This may also be referred to as the heat exchange rate, which is determined by
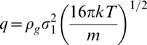
(20)The momentum of a particle after collision with a ghost particle at *t_u_* is selected randomly from a Boltzmann distribution of values at temperature *T*.

### Calculation of the equilibrium properties of the one-dimensional protein model using DMD

Since the protein model introduced here is described by a step-wise potential, its implementation is straightforward given the nature of DMD. We examine the effect of macromolecular crowding in DMD by inserting crowders that are purely modeled as repulsive hard sphere potentials. The hard sphere repulsion distance between a crowder and protein “subunit” is modeled as
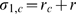
(21)The radius of the spherical protein subunit is defined as 13 Å for all simulations. Values for the crowder radius (*r_c_*) were determined according to a specified ratio relative to the size of the subunit and remain fixed throughout a given simulation.

All simulations are carried out using an Andersen thermostat [49] set at a reduced temperature of 1.0 *ε*/*k* with ghost particles that account for less than 1% of the occupied volume [31]. Prior to the equilibrium simulations, a relaxation step is performed to introduce crowding reagents into the system while alleviating potential clashes. The relaxation step employs a temporary soft potential that steadily increases the distance between crowders and the protein until the hard sphere repulsive distance is reached. To ensure adequate sampling of each system, all equilibrium simulations are run for 1×10^6^ time units.

For a given simulation of *n* trajectories, the fraction of model protein in the native state is
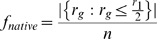
(22)where the numerator represents the number of elements in the set of *r_g_* in which *r_g_* is equal to or less than half of *r_1_*. Histograms were generated to determine equilibrium values of *P(r)*.

## Results

### Stability of the protein model as a function of urea concentration in dilute solution

**Figure 2:**
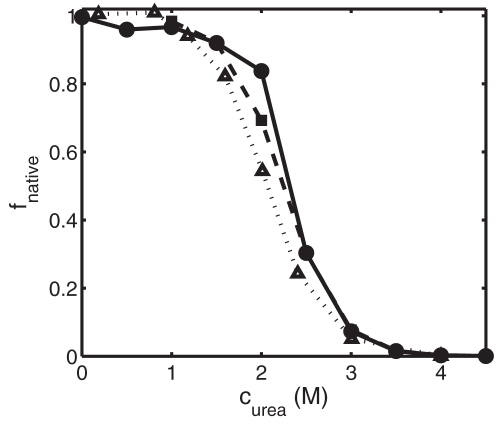
Equilibrium fraction of native conformation plotted as a function of *c_urea_*. Solid circles: Calculated using DMD. Open squares: calculated using Eq. 4 with *U = U_o_*. Triangles: experimentally measured dependence for Ribonuclease A, as reported by Tokuriki et al [39].

The dependence of *f_native_* upon urea concentration calculated using Eq. 4 with *U* = *U_o_*, and calculated using DMD, are plotted in Fig. 2. For comparison, the experimentally measured fraction of native ribonuclease A is also plotted as a function of urea concentration [39].

**Figure 3:**
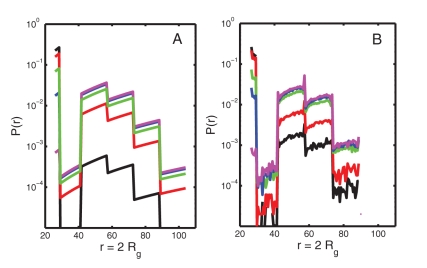
Equilibrium values of *P(r)* at different urea concentrations. Plotted as a function of *r* according to Eq. 1 (A) and DMD (B). *c_urea_* = 1 M (black), 2 M (red), 2.5 M (green), 3 M (blue), 4 M (magenta).

Distributions of *r*, calculated as a function of *c*_urea_ according to Eq. 1 with *U* = *U_o_* (Fig. 3 *A*) and calculated using DMD (Fig. 3 *B*), are plotted on a logarithmic scale. Generally good agreement is obtained between the calculated probabilities for *P*>0.001. It is likely that states with *P*<∼0.001 are relatively rarely observed during the DMD simulation and subject to stochastic errors of estimate. However, since such low probability states contribute little to the equilibrium average properties of the system, significant fractional errors of estimate of the probability of these states (which are magnified on the logarithmic scale) do not result in substantial errors in calculated equilibrium properties.

### Effect of hard sphere crowding upon stability of protein model

**Figure 4:**
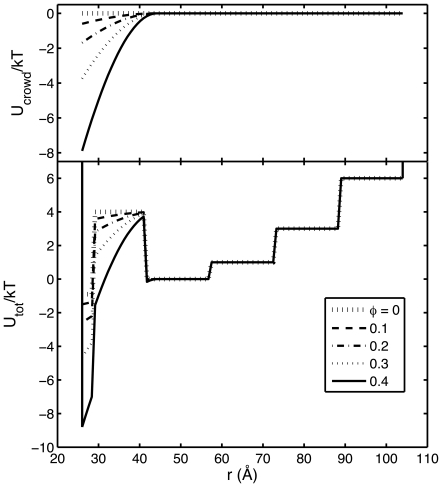
Effect of potential of average force exerted by crowders on the total potential. *U_crowd_* [upper panel] and *U_tot_* [lower panel] plotted as a function of *r*, calculated for *c_urea_* = 3 M and *R_c_* = 0.7 as described in text, for 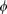 = 0 (large dotted line), 0.1 (dashed line), 0.2 (dot-dashed line), 0.3 (small dotted line), and 0.4 (solid line).

**Figure 5:**
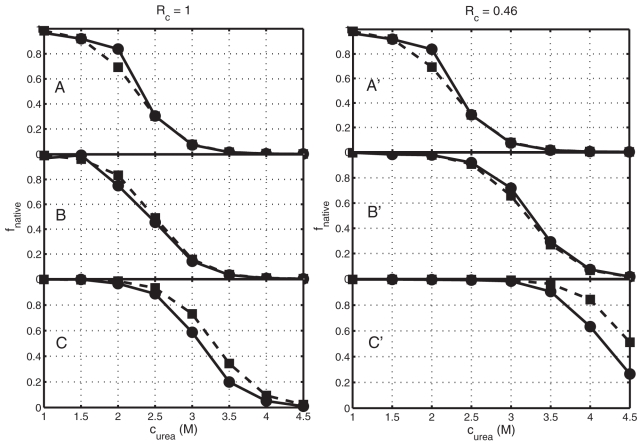
Urea denaturation curves for different crowder sizes. *f_native_* plotted as a function of *c_urea_* using DMD (circles) and the statistical-thermodynamic model (squares) for *R_c_* = 1 (panels A,B,C) and 0.46 (panels A′,B′,C′), and 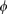 = 0 (panels A, A′), 0.2 (panels B, B′), 0.3 (panel C′), and 0.4 (panel C).

**Figure 6:**
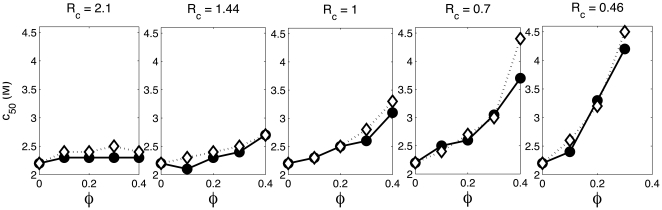
Half-denaturation urea concentration plotted as a function of 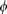. Calculated using DMD simulations (circles) and the statistical-thermodynamic model (diamonds) for *R_c_* = 2.1, 1.4, 1, 0.7, and 0.46.

The crowding-induced potential of mean force, *U_crowd_(r)*, calculated as described above, is plotted for *R_c_* = 0.7 and various values of fractional volume occupancy 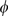 in the top panel of Fig. 4. Corresponding values of *U(r)* are plotted in the lower panel. It may be seen that the crowding-induced potential is short-ranged, has the largest influence on the potential in bin 1, a smaller effect on the potential in bin 2, and essentially no effect on the potentials in bins 3–6. Comparing Figs. 1 and 4, it appears that the major effect of adding crowder to the solution is to lower the potential in bin 1 in a fashion that, to a first approximation, counteracts the effect of urea in raising this potential. We thus expect added crowder to stabilize the native state with respect to urea-induced unfolding. The dependence of the fraction of native state upon urea concentration, as calculated according to the statistical-thermodynamic model and according to DMD simulations, are plotted in Fig. 5 for two different values of relative crowder size and different values of 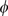. The magnitude of the overall effect of crowding may be quantified by the parameter *c_50_*, the urea concentration required to induce half of the protein to unfold at equilibrium. The dependence of *c_50_* upon 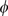, calculated according to the statistical-thermodynamic model and DMD simulations is plotted in Fig. 6 for all values of *R_c_* at which DMD simulations were carried out. Generally good agreement between the approximate theory and the simulations is obtained.

## Discussion

Although the discretized one-dimensional potential of average force specified in Table 1 was designed to qualitatively imitate features of a real protein, ribonuclease A, as shown in Figs. 1 and 2, we emphasize that this model is not meant to physically represent the actual process of protein folding. The purpose of the model is to attain insight into the effect of crowding on the intramolecular associations underlying conformational isomerization in a model protein using a simplified theory and DMD simulations. Our model provides didactic value such that the most fundamental elements of protein folding, namely the interplay between short-ranged and long-ranged interactions, are retained at a simplistic level to enable exploration of the features of macromolecular crowding using statistical thermodynamic theory.

From this model, we find that results obtained from analytical treatment of the system are quantitatively similar to DMD results. Both the analytical solution and the simulation indicate that the degree of stabilization of the native state of the protein afforded by the presence of a given volume fraction of inert hard particle crowder is strongly dependent upon the ratio of the size of the “protein” to the size of the crowder, increasing as crowder size decreases. This conclusion follows qualitatively from simple principles of volume exclusion [12], [13], [51], has been predicted on the basis of earlier excluded volume treatments [14], [15], [52], and demonstrated experimentally [53]. The model presented here provides another quantitative estimate of the magnitude of the effect of crowder size, which will be characterized more fully in a subsequent study using a more detailed protein model.

It has been observed that the magnitude of the crowding effect on an isomerization reaction (such as protein folding) increases with extent of isomerization-linked change in the ratio of co-solute accessible surface to volume [12]. Thus the effect of crowding upon the present one-dimensional model, in which folding is represented by tangential contact between two spheres, is expected to be smaller than the effect of crowding on a real protein folding reaction, since the latter would resemble a unimolecular condensation of an extended polypeptide chain, corresponding to a much larger fractional reduction in co-solute accessible surface area.

The dependence of the stability of our simplified protein model upon urea concentration that is predicted by the analytical model agrees reasonably well with that obtained from the DMD simulation except at the lowest values of *R_c_* and the highest values of 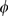. This overall agreement arises because within the context of the two-state model, protein stability is determined by the relative free energies of the “native state” (i.e., configurations in bin 1) and the “non-native state” (i.e. configurations in bins 2–6). The analytically calculated crowding potential is in good agreement with the PMF obtained from the DMD simulation at distances close to the contact distance, that is, within bin 1. However, it underestimates the magnitude of the crowding potential at larger distances, and so leads to an underestimate of the relative stability of conformations in bin 2 most strongly and to a lesser extent in bin 3. However, since conformations in bin 2 are defined as intrinsically high energy (*U_o_* = 4 *kT*) and hence extremely poorly populated in the absence of crowding (see Fig. 3), only a very large lowering of the free energy of these conformations (greater than about 3–4 *kT*) will increase the equilibrium population of conformations in these bins to the point at which they contribute significantly to the total Boltzmann-weighted average free-energy of the “non-native” state.

The model presented in this study enables a quantitative comparison of modeling the effect of chaotropes on protein folding using either explicit crowders via simulations or modeling implicit crowders as a potential of mean force. Here it is demonstrated that quantitative agreement can be obtained between scaled particle theory and DMD simulations, at least to the extent that such equilibria may be modeled as intramolecular association reactions. Furthermore, the simple model presented provides didactic value in that even by reducing protein folding to its most basic elements, we can gauge the effect of crowders on promoting isomerization.
